# High frequency oscillations in the intra-operative ECoG to guide epilepsy surgery (“The HFO Trial”): study protocol for a randomized controlled trial

**DOI:** 10.1186/s13063-015-0932-6

**Published:** 2015-09-23

**Authors:** Maryse A. van ’t Klooster, Frans S. S. Leijten, Geertjan Huiskamp, Hanneke E. Ronner, Johannes C. Baayen, Peter C. van Rijen, Martinus J. C. Eijkemans, Kees P. J. Braun, Maeike Zijlmans, on behalf of the HFO study group

**Affiliations:** Department of Neurology and Neurosurgery, Brain Center Rudolf Magnus, University Medical Center Utrecht, PO Box 85500, 3504 Utrecht, GA The Netherlands; Department of Clinical Neurophysiology and Magnetoencephalography Center, VU University Medical Center, Amsterdam, The Netherlands; Neurosurgical Center Amsterdam, VU University Medical Center Amsterdam, Amsterdam, The Netherlands; Department of Child Neurology, Brain Center Rudolf Magnus, University Medical Center Utrecht, Utrecht, The Netherlands; Julius Center for Health Sciences and Primary Care, University Medical Center Utrecht, Utrecht, The Netherlands; SEIN-Stichting Epilepsie Instellingen Nederland, Heemstede, The Netherlands

**Keywords:** Epilepsy surgery, Intra-operative electrocorticography, ECoG, Pediatric epilepsy, Epileptogenic zone, High frequency oscillations, HFOs, Randomized controlled trial

## Abstract

**Background:**

Intra-operative electrocorticography, based on interictal spikes and spike patterns, is performed to optimize delineation of the epileptogenic tissue during epilepsy surgery. High frequency oscillations (HFOs, 80–500 Hz) have been identified as more precise biomarkers for epileptogenic tissue. The aim of the trial is to determine prospectively if ioECoG-tailored surgery using HFOs, instead of interictal spikes, is feasible and will lead to an equal or better seizure outcome.

**Methods\Design:**

We present a single-blinded multi-center randomized controlled trial “The HFO Trial” including patients with refractory focal epilepsy of all ages who undergo surgery with intra-operative electrocorticography. Surgery is tailored by HFOs (arm 1) or interictal spikes (arm 2) in the intra-operative electrocorticography. Primary outcome is post-operative outcome after 1 year, dichotomized in seizure freedom (Engel 1A and 1B) versus seizure recurrence (Engel 1C-4). Secondary outcome measures are the volume of resected tissue, neurologic deficits, surgical duration and complications, cognition and quality of life. The trial has a non-inferiority design to test feasibility and at least equal performance in terms of surgical outcome. We aim to include 78 patients within 3 years including 1 year follow-up. Results are expected in 2018.

**Discussion:**

This trial provides a transition from observational research towards clinical interventions using HFOs. We address methodological difficulties in designing this trial. We expect that the use of HFOs as a biomarker for tailoring will increase the success rate of epilepsy surgery while reducing resection volume. This may reduce neurological deficits and yield a better quality of life. Future technical developments, such as validated automatic online HFO identification, could, together with the attained clinical knowledge, lead to a new objective tailoring approach in epilepsy surgery.

**Trial registration:**

This trial is registered at the US National Institutes of Health (ClinicalTrials.gov) #NCT02207673 (31 July 2014) and the Central Committee on Research Involving Human Subjects, The Netherlands #NL44257.041.13 (18 March 2014).

## Background

The current success rate of epilepsy surgery in patients with refractory focal epilepsy lies between 36 and 84 % after 1 year [1]. Intra-operative electrocorticography (ioECoG) can be performed during surgery to optimize delineation of the epileptogenic area by taking into account interictal spikes or spike patterns. This so called “tailoring” affects surgical decision-making [2]. Resection of areas with interictal spikes has been associated with seizure freedom [3–5], whereas remaining spikes after resection have been suggested to indicate poor surgical outcome [2, 3, 6], while other studies contradict this [7–9]. The value of tailoring by interictal spikes can be questioned as they represent the irritative zone rather than the seizure onset zone [6], spikes can spread into non-epileptic surrounding areas [6], and can also arise from surgical manipulation [10]. This leaves tailoring based on spikes in the ioECoG under international debate. Incomplete resection of so called “ictiform spike patterns”, consisting of recruiting patterns, repetitive bursting patterns or continuous rhythmic spiking, has been reported to predict poor outcome [11].

High frequency oscillations (HFOs, 80–500 Hz) are proposed as a new and more precise biomarker for epileptogenic tissue than spikes [12–20]. HFOs are an indicator of the seizure onset zone [15]. Removal of tissue with HFOs, especially fast ripples (FR, 250–500 Hz), is linked to good surgical outcome [16, 18, 21, 22]. The area showing HFOs usually overlaps with, but is smaller than the irritative zone showing spikes [15]. HFOs mirror epileptic disease activity as they are prominently found in focal cortical dysplasia (FCD), with increased numbers in the more epileptogenic FCD type 2 compared to type 1 [17], and they increase when anti-epileptic drugs are tapered [23]. HFOs can be recorded during surgery, after anesthetics are reduced [24].

Two decades after their first discovery there is a rising demand to know whether HFOs can be used in clinical practice and decision-making [14, 25, 26]. Tailoring based on HFOs in the ioECoG could improve the chance of seizure freedom. Other beneficial aspects could be a smaller resected area, with less chance of neurological deficits, which – together with an equal or increased seizure freedom rate – may lead to an overall better quality of life. Last but not least, the identification of epileptic HFOs can be automated [12, 27–30] and, therefore, could yield a future objective approach that is easily implemented.

“The HFO Trial” is a randomized controlled trial (RCT) designed to investigate the feasibility and safety of using HFOs during intra-operative tailoring of epilepsy surgery. The primary objective of this RCT is to validate if tailoring based on HFOs versus interictal spikes in the ioECoG during surgery will lead to the same post-surgical seizure outcome 1 year after surgery (non-inferiority design). Secondary objectives are comparison of the volume of resected tissue, the duration of the surgery, the occurrence of complications and neurologic deficits, and changes in cognitive functioning and quality of life. RCTs in the field of epilepsy surgery are not trivial undertakings [31, 32]. We report the details of our study design, and share our considerations for the trial set-up.

## Methods

### Trial design

“The HFO Trial” is a single-blinded randomized controlled Dutch multi-center clinical trial. This trial is based on a non-inferiority design with an allocation ratio of 1:1. We chose a non-inferiority design, as we considered the primary objective of the study, to demonstrate that the intra-operative prospective use of HFOs to tailor surgery is feasible and will not lead to worse outcome than the widely applied method based on spikes. Secondary objectives of this study are to investigate whether the HFO-based, compared to spike-based, tailoring will lead to differences in the volume of resected tissue, neurologic deficits, surgical duration and complications, cognition and quality of life.

### Participants

Participants are candidates for epilepsy surgery who are referred to the Dutch Collaborative Epilepsy Surgery Program (DCESP) and are selected to undergo epilepsy surgery with ioECoG-based tailoring. Members of the DCESP come from the two Dutch epilepsy centers and three Dutch university medical centers performing epilepsy surgery. The two participating surgical centers in this trial are: 1) the UMC Utrecht, specialized in pediatric epilepsy surgery with 75 % of the patients being < 18 years of age at surgery, and 2) the VUmc Amsterdam, that operates on adult patients only (≥18 years). Patients and parents or care givers will be asked to participate if they comply with the following eligibility criteria:

1. Refractory focal epilepsy, defined as at least 2 seizures in the past 24 months, in spite the use of 2 or more different anti-epileptic drugs (AEDs).
2. Epilepsy surgery planned with ioECoG to tailor the resection. Note that in the participating centers, this automatically excludes standard right-sided temporal resections, disconnections and hemispherectomies in which no ioECoG is performed.
3. Command of the Dutch language by the patient or parents\legal representatives and capability of completing the Dutch questionnaires.
4. Able to give informed consent.

Exclusion criteria are:

1. Previous chronic ECoG (grid) monitoring preceding epilepsy surgery. This is a biased population, because results of the extensive pre-surgical work-up and the results of the monitoring period determine the outline of the resection. This includes precise knowledge of the seizure onset zone, the inter-ictal spikes and HFO areas.
2. Patients with an occipital focus undergoing ioECoG. Physiological FRs have been described to occur in the occipital lobe [33]. We deemed it unsafe to perform HFO-guided resections in these patients, as it is still difficult to discriminate between pathological and physiological HFOs.

### Interventions

Participating patients who will undergo ioECoG-tailored surgery will be randomized into an HFO-guided (arm 1) or standard epileptiform spikes-guided (arm 2) resection.

The ioECoG is routinely recorded at a sampling frequency of 2048 Hz using clinical electroencephalogram (EEG) software. A dedicated HFO team, including two experienced HFO observers, will perform the analysis during surgery when the patient has been allocated to the HFO arm. The HFO reviewers need to achieve consensus about the HFOs and distinguish them from artifacts. This team will, together with the clinical neurophysiologist in charge, advise the neurosurgeon on the extent of the tissue to be resected. HFO analysis is performed visually and off-line using Stellate Harmonie Reviewer (v7.0, Montreal, QC, Canada), as this is currently the only clinical EEG software that provides HFO visualization and marking. A split screen modus will be used to simultaneously visualize ripples (settings: finite impulse response (FIR) filter > 80 Hz, gain 5 μV/mm) and fast ripples (settings: FIR filter > 250 Hz, gain 1 μV/mm) at an elongated time interval of 0.4 s/page, according to the settings described in other studies [15, 18, 21, 22]. Based on pilot data, we expect that the visual analysis of HFOs will require 50 % more time compared to the visual analysis of epileptogenic spikes, which usually takes 3 to 5 minutes per recording. This is an intention-to-treat study. HFOs in eloquent regions will not be resected, similar to standard tailoring practice based on spikes. In absence of HFOs, ictiform spike patterns (for definition see introduction) [11] will always be resected, irrespective of treatment allocation. If no HFOs or ictiform spikes are found a planned resection is performed according to size and location of epileptogenic structural lesion(s), similar to standard clinical practice when no spikes could be identified.

### Outcomes

Primary outcome parameter is the post-surgical seizure outcome after 1 year based on the Engel classification [34]. Seizure outcome will be dichotomized in seizure freedom (Engel 1A and 1B) versus seizure recurrence (Engel 1C-4). We decided to include possible auras (Engel 1B) in the seizure-free group, because it can be difficult to distinguish true auras from aura-like non-epileptic sensations. Post-surgical outcomes at 6 to 8 weeks, 6 and 12 months will be determined by means of a follow-up questionnaire completed by the patient or legal representatives. So called “running down” seizures, seizures occurring only during the first 2 weeks after surgery are not considered as seizure recurrence.

Secondary outcome parameters are:

1. Volume of resected tissue (in cm^3^): the volume of resected tissue is determined by voxel-based volumetrics of the routine pre-surgical and post-surgical 3D whole head T1 magnetic resonance imaging (MRI) scan.
2. Neurological deficits: neurological deficits are assessed by neurological examination and translated into the National Institutes of Health Stroke Scale (NIHSS) before surgery and before hospital discharge after surgery. The cumulated NIHSS score ranges between 0 and 42; a difference of 1 point on the NIHSS scale between the 2 tests is considered clinically relevant. In that case the NIHSS will be repeated at follow-up moments. Additionally, neurological deficits will be classified as “pre-existing” (either improved or aggravated) or as “new” (either anticipated or unexpected).
3. Duration of surgery: post-hoc analysis of duration of surgery (start-stop time neurosurgeon, in minutes) and ioECoG recording time (in minutes).
4. Surgical complications: accounts are kept of the number of (post-) operative complications, such as bleeding, infection, unexpected or aggravated neurological deficits. These events will also be reported as (serious) adverse events ((S)AE).
5. Cognition: comparison of results from pre-operative and 12-month post-operative neuropsychological evaluation. This routine neuropsychological evaluation includes testing of IQ, working memory and processing speed. All tests performed are chosen according to the age of the patient, but report on the same domains. Per domain individual patients’ results will be dichotomized into negative, no, or positive change compared to pre-surgical baseline.
6. Quality of life (QoL): QoL will be determined pre-operatively, and post-operatively after 6 to 8 weeks, and 6 and 12 months using a visual analog scale (VAS) on overall self-perceived quality of life, or by the parents in case of children < 12 years of age.

**Fig. 1:**
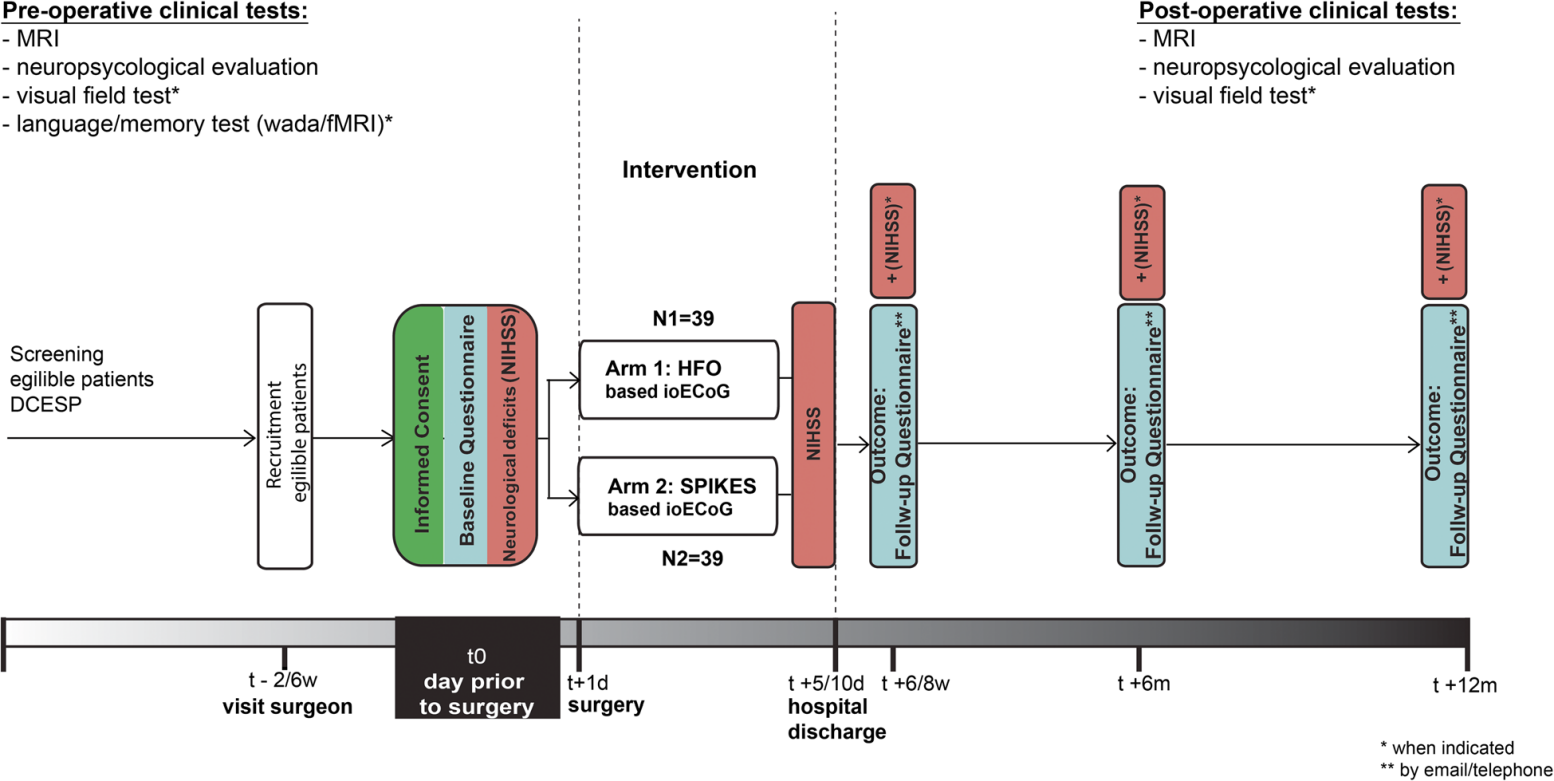
Timeline of the study procedures. On the day prior to surgery, the patient signs the informed consent form and subsequently the baseline questionnaire and neurological examination (by means of National Institutes of Health Stroke Scale (NIHSS)) are completed and the patient is enrolled in the study by randomization. Note that the follow-up questionnaire collects information about (preliminary) outcome, anti-epileptic drug use, quality of life and occurrence of (serious) adverse events. Additional information is collected from routine clinical tests that are performed during the pre-surgical and post-surgical period

Data collection, management and storage is done in open source clinical trial software OpenClinica (OpenClinica, LLC, Waltham, MA, USA; www.openclinica.com), managed by the IT division of the Neurology and Neurosurgery Department of the UMC Utrecht. Figure 1 shows the timeline of the study procedures.

**Fig. 2:**
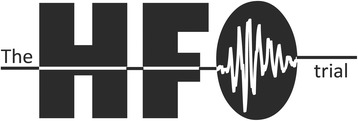
Logotype “The HFO Trial”

### Sample size

The sample size calculation is based on a success rate of surgery (defined as Engel 1A or 1B at 1 year) in the control group (tailoring based on spikes) of 65 %, and the expected success in the experimental group (HFOs) of 80 %. The resulting margin is 15 %. Using a 1-sided 95 % confidence interval and a non-inferiority limit of 10 %, to acquire an 80 % power we need 39 patients per group. This results in a total of 78 patients. We anticipate that 80 % of the eligible patients will participate in the study.

We expect the potential loss-to-follow-up after the initial intervention to be low. Routine clinical visits to the neurosurgeon and neurologist are scheduled after surgery at 6 to 8 weeks, 6 months and 12 months. Our FU questionnaires coincide with these visits. Inclusions will proceed until the target sample size is achieved to address loss to follow-up. Loss to follow-up can be expected from; 1) withdrawal by the subject from the study during follow-up before the first preliminary outcome determination at 6–8 weeks, and 2) withdrawal from the study during the surgery by the surgeon or dedicated team due to urgent medical or technical issues.

Subjects are replaced if they withdraw from the study during follow-up before the first preliminary outcome determination at 6–8 weeks. Subjects who are withdrawn from the study during the surgery by the surgeon or dedicated team due to urgent medical or technical issues are also replaced.

### Interim analysis

Safety and efficacy analysis will be performed by the statistician of the independent Data Monitoring Committee (DMC) after the first 20 and 40 included patients. This analysis will be based on the available, preliminary, post-surgical outcomes and also includes the number of surgical complications and (serious) adverse events ((S)AE). The DMC can advise on premature termination of the study in case of harmful effects, or superiority, in one of the treatment arms or in case of non-feasibility issues.

### Randomization

Eligible epilepsy surgery patients and/or parents or caregivers will be informed about this study by the researcher in person, minimally 2 weeks in advance of the surgery. Informed consent is asked on the day prior to the day of surgery. After written consent, the patient will be randomized into the treatment allocation (ALEA version-release 2.2). Stratification for participating site and epilepsy type (extra-temporal versus temporal lobe epilepsy) is performed using block randomization of 2, 4 and 6 patients. The stratification for participation site includes an indirect stratification for age, as in the VUmc only adults and in the University Medical Center Utrecht (UMCU) predominantly children are operated on.

### Blinding

Blinding of the treating physicians and neurosurgeons for treatment allocation is not feasible because of the character of the intervention. Therefore, this is a single-blinded trial as patients will be blinded for the treatment allocation to avoid bias of the follow-up results. To guaranty the blinding of the patient, the outpatient physicians, neurologist and psychologist involved in the follow-up procedures are blinded for the ioECoG report during the entire period of follow-up. After study completion or termination patients who wish so will be de-blinded.

### Statistical methods

The primary “intention-to-treat” analyses at study completion are based on the difference between the treatment arms with respect to surgical outcome after 12 months. The primary outcome is a categorical outcome, seizure freedom versus seizure recurrence. Risk ratios (RR) and risk difference with 95 % confidence interval will be calculated (*X*^2^- test, 1-sided). Secondary outcomes are the amount of tissue resected, neurological deficits, duration of surgery, complications, cognition and quality of life. For the secondary outcome parameters consisting of continuous variables, a *T*-test with 95 % confidence interval is calculated and tested (1-sided). For the secondary outcome parameters consisting of categorical variables, the RR and risk difference with a 95 % confidence interval are calculated and tested using a Chi-square test (1-sided). Logistic regression analysis will be performed: 1) to adjust for the stratification factors, by including site and epilepsy type as variables in analysis, and 2) to investigate if there are relations between seizure outcome and subject variables, such as age, gender, and pathology, or with experimental variables, such as the operating surgeon and the amount of anesthesia received. Note that we are not going to use a mixed model to adjust for site, as we consider two sites too small a sample to be representative for other sites. Regression analysis is also performed between seizure outcome and the secondary outcome parameters.

In the logistic regression analysis we will, after screening of the number of missing data, perform multiple imputation. For the primary and secondary endpoint no imputation will be performed. Statistical analysis will be performed in SPSS version 21 (SPSS Inc., Chicago, IL, USA) or higher and/or R version 3.1 or higher.

The number of eligible patients withholding consent will be registered, and demographic information will be collected anonymously for post-hoc explorative analysis to understand potential bias in study outcomes.

### Safety aspects

Adverse events (AEs) are defined as any undesirable experience occurring to a subject during the study, whether or not considered related to the experimental treatment. A SAE is defined as any untoward medical occurrence or effect that results in death, is life-threatening, requires (prolonged) hospitalization, results in persistent or significant disability or incapacity, or is a new event of the trial that is likely to affect the safety of the subjects. We will not report the majority of direct postsurgical complaints, such as nausea, headache, abdominal pains or pain related to the surgical scar during the hospitalization period, as AEs, as those result directly from brain surgery and will resolve before discharge. Similarly, direct post-operative functional deficits, due to surgically induced cerebral edema, will often resolve prior to discharge; these will not be reported as (S)AE. SAEs will be closely monitored by the researchers within the timeframe of hospital admission (normal range ≤ 10 days after surgery). The neurological deficits will be assessed with the NIHSS questionnaire (see also neurological deficits). During the 1 year follow-up period all reported AEs and SAEs, independent of the site, will be registered by the researchers and reported to the DMC. SAEs are reported to the Medical Ethics Committee.

### Ethical considerations

Informed consent will be obtained from all participants and/or their legal representative(s), in writing, before inclusion in the trial. “The HFO Trial” protocol has been approved by the Medical Ethics Committee of the UMC Utrecht (MEC-13-389). “The HFO Trial” is registered at the US National Institutes of Health (ClinicalTrials.gov) #NCT02207673 and the Central Committee on Research Involving Human Subjects, The Netherlands #NL44257.041.13.

## Discussion

We announce, by reporting our study design, the start of the “The HFO Trial,” a multi-center RCT in epilepsy surgery. This is the first clinical trial investigating tailoring based on biomarkers in the ioECoG, i.e. HFOs versus interictal spikes, with respect to post-surgical seizure outcome.

### Trials in epilepsy surgery

RCTs are considered the “gold standard” for evaluating therapeutic interventions, but surgical RCTs are challenging and only few have successfully been completed in the field of epilepsy surgery. So far 13 RCTs were performed in the period 1992–2012, and 5 of them, all in temporal lobe resections, investigated a new surgical strategy or compared AED prescription versus (early) surgery [31, 35]. Currently, five ongoing RCTs in the field of epilepsy surgery are registered on the international trial register “clinicaltrials.gov.” Two of these RCTs investigate a new surgical strategy, including the trial reported here.

### Considerations

An important consideration in RCTs in epilepsy surgery is feasibility, which is influenced by the (in)ability of recruiting enough eligible patients with sufficient speed. The difficulty to standardize diagnostic testing, medical treatment and surgical interventions across multiple centers is of great influence [31, 32, 35]. In the Netherlands, all epilepsy surgery is performed in three medical centers and is embedded in the Dutch Collaborative Epilepsy Surgery Program (DCESP) [36]. Cases are discussed within this national team of (pediatric) neurologists, clinical neurophysiologist, (pediatric) neuropsychologists, nurse practitioners, physician assistants, radiologists and surgeons. This unique collaboration secures more or less standardized protocols and consistent indications for eligibility and treatment. The small distance (<50 km) between the 2 centers in Amsterdam and Utrecht gives us the opportunity to have 1 dedicated HFO team to execute the trial in both centers, including the HFO analysis during surgery.

We chose post-surgical outcome as primary outcome parameter, as the ultimate goal of epilepsy surgery is to achieve seizure freedom. This is the most relevant clinical parameter as the number of seizures and the side effects of AED use are the main determinants of QoL in epilepsy patients [1]. We chose to differentiate between seizure freedom ± auras (good outcome) and seizure recurrence (poor outcome) although in the literature, good outcome is often defined as Engel 1 and 2 [15, 16]. We deliberately applied this stricter definition, because we aim to find and remove all epileptogenic tissue based on HFOs and thus aim for seizure freedom as completely as possible. We included auras in the good outcome group, because distinction of the presence or absence of aura sensations in the first year after surgery, while still using AEDs, may be subjective. We will add post-hoc analyses using other dichotomies of the outcome score (for example, Engel 1A versus all others). These analyses might help to design future prospective studies. Recent studies have suggested that early AED withdrawal does not affect long-term seizure outcome or cure. It might unmask incomplete surgical success sooner [37]. Definite proof of complete removal of potentially epileptogenic tissue requires enduring seizure freedom over many years without the use of AEDs. We will collect information about AED use during follow-up for post-hoc analysis.

Previous epilepsy surgical RCTs included patients with intractable epilepsy with a minimal age of 12 years [31]. Including children in clinical trials is inextricably connected to ethical concerns [26]. A shift in epilepsy surgery towards the pediatric population has, however, been set in motion over the last years and the proportion of young children who undergo epilepsy surgery is increasing [36]. Children are considered for early epilepsy surgery nowadays because successful resection of epileptic foci may lead to seizure freedom and medication freedom, and may improve social, psychological and cognitive development [38, 39]. We chose to include children, irrespective of age, because the research question about seizure outcome and the expected smaller resection that might reduce neurological and cognitive deficits are most relevant in this population. Limiting inclusion solely to adults would not only provide a lower number of eligible patients for participation, but would exclude the patient population who might benefit most.

We chose for a non-inferiority design to support feasibility of the trial, although literature findings suggest that HFOs are the superior biomarker for epileptogenic tissue rather than spikes [12, 13, 15, 16, 18–21, 29, 28]. The limiting factor of a superiority design is the large sample size needed, which would require participation of more than two centers. Currently, the few HFO experts and the fact that the surgeries and HFO analyses are time consuming limit the number of participating centers and patients. We consider the most important aspect of the study to assess the feasibility of using HFOs for tailoring. We think it is also important, at the same time, that automated ways of analysis are developed and implemented [27, 30], since these are indispensable for clinical application in other centers. Together with this trial this could set the stage for a larger, international RCT aimed at proving actual superiority of HFOs.

Our power calculation is based on estimated success rates deduced from retrospective studies up until 2013. These studies consisted of different patient populations. The current success rate of epilepsy surgery based on the old treatment, tailoring based on spikes, lies around 65 %; in resections for temporal lobe epilepsy (TLE) 60–90 % of patients achieve seizure freedom while in extra-temporal epilepsy it is around 40–65 % [40, 41]. We expected an increase in success percentage of 15 % leading to 80 % for HFOs based on retrospective studies in which part of the patients, with surgery based on spikes, had incomplete removal of HFO tissue and poor outcome [18], while incomplete removal of spikes does not predict seizure recurrence [15, 16]. We are aware that we chose a relatively large effect size. An overestimation of the success rate for the outcome in the HFO arm could lead to non-significant finding due to too a small population. Before we started with this RCT, we retrospectively studied our own ioECoG data in order to validate previous findings [22]. We found good outcome (Engel 1A and 1B after 1 year) in 70 % of patients and tailoring based on FRs could have improved outcome in 15 % of these patients [22].

This study also gave rise to the question whether or not we need to discriminate between epileptic and physiological HFOs, as we found some FRs, although far away from the area of resection and in functionally eloquent areas such as the sensorimotor and Broca’s areas, after resection in two patients who were seizure-free and medication-free. This means that we have to be careful when assessing HFOs in eloquent areas. In our retrospective data analysis we found that residual FRs were strongly related to epileptic tissue and poor outcome, while for ripples we found no association [22].

Tailoring based on spikes in the ioECoG is not an evidence-based method, as no RCT has compared tailoring to resection without ioECoG, but we relate to it as the “gold standard” [6]. The same restrictions for tailoring are valid for HFOs as for spikes. The ioECoG might either confirm the surgical plan or change the surgical plan, but reduction or extension of the planned resection might be limited by eloquent cortex or anatomy. Events, spikes or HFOs, occurring remote from the resection site are usually not considered indicative for removal. Upfront it may be hard to tell if tailoring will really influence the surgical plan, even though in our population we aim to perform ioECoG only with a valid indication. We will, prospectively, collect data on the influence of events on the actual clinical decision and perform a post-hoc analysis to clarify in which patients tailoring actually changed the surgical plan.

The visual analysis of HFOs requires experienced observers, and additional analysis time due to the offline analysis and expanded time settings for ECoG display. We estimate this additional analysis time is 5 to 10 minutes per ioECoG recording, compared to spike analysis. A disadvantage of using spikes is that they have a lower inter-observer agreement compared to HFOs [42]. An associated risk of the use of HFOs is undersampling and underdetection of HFOs compared to spikes, as HFOs are a more local phenomena than spikes [15], and the negative effect of anesthetics on the number of HFOs [24]. These concerns are preempted similar as for the procedures during tailoring based on spikes; multiple recordings are made before and after resection, where the sampling strategy is based on the results in previous recordings to guaranty optimal sampling. All recordings are made while tapering the propofol until a continuous EEG pattern can be seen.

### Expected benefit

We believe that an RCT is the proper way to prospectively test the beneficial properties of HFOs as a new biomarker for delineation of epileptogenic tissue and eventually improve the success rate of epilepsy surgery. Retrospective research suggests that HFOs are more specific and precise biomarkers for the epileptogenic zone than spikes. Complete resection of the epileptic focus may lead to seizure freedom and medication freedom, and may improve social, psychological and cognitive development, especially in children. Potential benefits from a smaller or more precise resection would be reduced neurological deficits and, combined with equal or better seizure outcome, should improved quality of life. The identification of HFOs can be standardized and automated and thereby potentiate an objective tailoring approach for international implementation.

## Trial status

The first patient was enrolled in “The HFO Trial” on 6 November 2014 in the UMC Utrecht. Figure 2 shows the logotype of the “The HFO Trial”. At the moment of acceptance of this manuscript, August 2015, in total 10 patients have been included. Enrollment at the second site, VUmc Amsterdam, will start in autumn 2015. Results are expected in 2018.

## Abbreviations

3D: three-dimensional
AE: adverse event
AEDs: anti-epileptic drugs
DCESP: Dutch Collaborative Epilepsy Surgery Program
DMC: Data Monitoring Committee
EEG: electroencephalogram
FCD: focal cortical dysplasia
FIR: finite impulse response
FRs: fast ripples (250–500 Hz)
HFOs: high frequency oscillations (80–500 Hz)
ioECoG: intra-operative electrocorticogram
MRI: magnetic resonance imaging
NIHSS: National Institutes of Health Stroke Scale
QoL: quality of life
RCT: randomized controlled trial
RR: risk ratio
SAE: serious adverse event
TLE: temporal lobe epilepsy
UMCU: University Medical Center Utrecht
VAS: visual analog scale
